# CDH1 is Identified as A Therapeutic Target for Skin Regeneration after Mechanical Loading

**DOI:** 10.7150/ijbs.51309

**Published:** 2021-01-01

**Authors:** Xiaolu Huang, Xiao Liang, Yiwen Zhou, Haizhou Li, Hengyu Du, Yinjun Suo, Wenhui Liu, Rui Jin, Bangda Chai, Ran Duan, Haizhou Li, Qingfeng Li

**Affiliations:** a Department of Plastic & Reconstructive Surgery, Shanghai Ninth People's Hospital, Shanghai Jiao Tong University School of Medicine, 639 Zhizaoju Road, Shanghai 200011, P.R. China.

**Keywords:** skin regeneration, tissue expansion, WGCNA, Mechanical stimuli

## Abstract

**Rationale:** Mechanical stimuli in the microenvironment are considered key regulators of cell function. Clinically, mechanical force (tissue expander) is widely used to regenerate skin for post-burn or trauma repair, implying that mechanical stretching can promote skin cell regeneration and proliferation. However, the underlying mechanism remains unknown.

**Methods:** Microarray analysis was utilized to detect the hub gene. The expression of Cdh1 as examined in cells and tissues by western blot, q-PCR and immunohistochemistry staining respectively. Biological roles of Cdh1 was revealed by a series of functional in vitro and in vivo studies.

**Results:** Microarray analysis identified *Cdh1* as a hub gene related to skin regeneration during rat cutaneous mechanical loading. In vitro studies suggested that both mechanical loading and *Cdh1* interference induced keratinocyte dedifferentiation and enhanced stemness, promoting cell proliferation and prevent apoptosis. Furthermore, the forkhead box O1/Krüppel-like factor 4 (FOXO1/KLF4) pathway was activated and contributed to the keratinocyte dedifferentiation. In vivo studies showed that mechanical loading and *Cdh1* interference facilitated epidermal dedifferentiation and promoted dermal collagen deposition, and that *Cdh1* overexpression could block such influence.

**Conclusions:** In this study, we show that E-cadherin (CDH1), a well-known cell-cell adhesion molecule, plays a crucial role in mechanical stretch-induced skin cell regeneration and proliferation. We have shown for the first time the process by which mechanical stress is transmitted to the epidermis and induces a downstream signaling pathway to induce epidermal cells to differentiate. These findings demonstrate that *Cdh1*-induced keratinocyte dedifferentiation is a crucial event in mechanical stretch-mediated skin regeneration and that *Cdh1* may serve as a potential therapeutic target for promoting skin regeneration.

## Introduction

Increasingly, mechanical forces in the microenvironment are being recognized as playing critical roles in regulating embryo development, tissue patterning, and cell behavior 1-5. Skin tissue is highly sensitive to mechanical loading 6 and can be expanded after stretching 7. Clinically, mechanical force is utilized widely to regenerate additional skin tissue for post-burn or trauma repair 8. While mechanical stretch increases epidermal keratinocyte proliferation 9, 10 and promotes skin regeneration 11, 12, the underlying cellular and molecular mechanisms have not been studied in-depth. Nevertheless, the stretch-induced skin regenerative capacity is limited (usually less than 2-3-fold of its original area) 7. Therefore, understanding the mechanism of mechanical stretch-induced skin regeneration and finding an effective drug target would be an effective means of resolving this bottleneck.

In this study, we explored the dominant factors of skin regeneration induced by mechanical stretch in Rat. We used weighted gene co-expression network analysis (WGCNA) 13, and locate the E-cadherin (CDH1) gene, which is recognized as the hub gene in the skin regeneration-related gene module after mechanical loading. Further, we determined that CDH1 knockdown could induce epidermal cell dedifferentiation and epidermal stem cell marker expression in vitro and in vivo and functioned via forkhead box O1/Krüppel-like factor 4 (FOXO1/KLF4) transcription factor (TF) activation and binding to the epidermal stem cell marker regulatory regions. In this context, we reveal the basic mechanism of skin tissue regeneration induced by mechanical loading and that CDH1 could serve as an important drug target for resolving the clinical bottleneck of skin regeneration.

## Results

### Clinical Example of Mechanical Loading-induced Skin Tissue Regeneration and Complications

**Fig 1:**
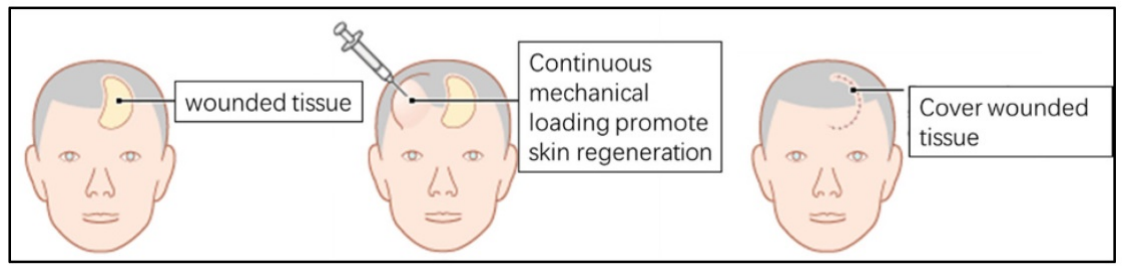
Diagram of clinical examples of mechanical loading-induced skin regeneration.

**Fig 2:**
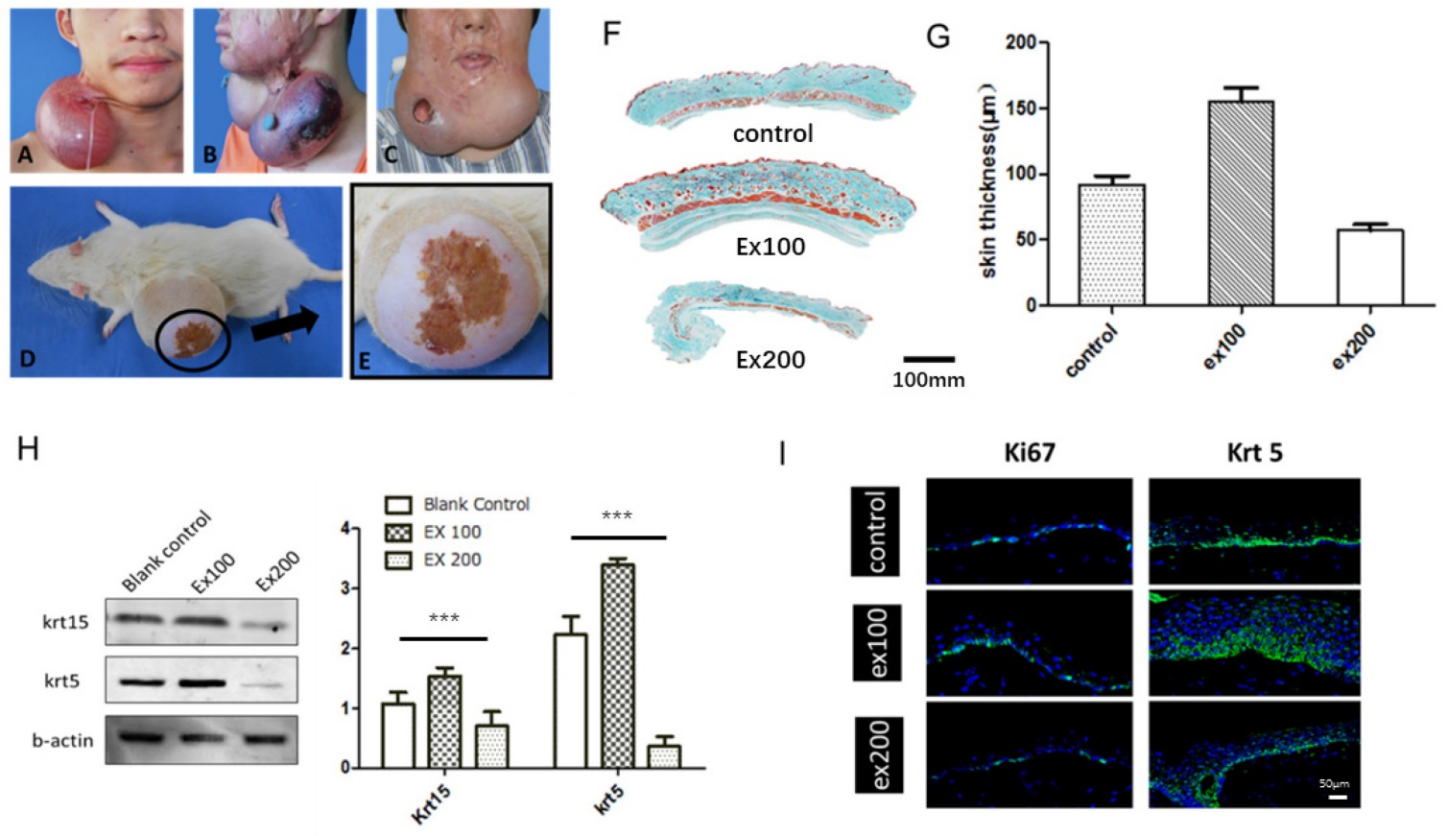
The relationship between skin regeneration and proliferation and expansion volume. (*A*) Skin overexpansion triggered flap thinning and capillarization in a patient. (*B*) Skin overexpansion caused flap necrosis in a patient. (*C*) Skin overexpansion led to ulcer formation in a patient. (*D*) Skin overexpansion caused flap necrosis in the rat model. (*E*) Enlarged image of flap necrosis in (*D*). (*F*) Masson trichome staining of rat skin tissue thickness following different expansion volume (ex100: 100-mL expansion, ex200: 200-mL expansion). Scale bar: 100 mm. (*G*) Rat skin thickness varied with expansion volume (ex100: 100-mL expansion, ex200: 200-mL expansion). (*H*) Western blot of epidermal stem cell markers in epidermis of rat skin expansion model. Blots were analyzed quantitatively using one-way ANOVA and multiple comparison: KRT15 (*P* < 0.0001) and KRT5 (*P* < 0.0001) expression were statistically different. (*I*) The normal or stretched skin from rats and humans were stained to demonstrate the changes in the proliferation marker Ki67 (rat model, green) and KRT5 (human, green), and DAPI (blue) Scale bar: 50μm. *N* =3 rats/group repeated 3-5 experiments.

Skin stretching is used for reconstructing skin tissue, such as post-burn/trauma scars, congenital deformities and skin defects after tumor resection. Clinically, a balloon-like inflatable implant (silicone expander) was used to regenerate skin tissues by continuous application of a mechanical load **(Fig. 1)**. However, the proliferation and regeneration capacity of skin tissue under mechanical stretch is not infinite, complications occur when human skin is over expanded **(Fig. 2A-C)**, a similar phenomenon was observed in the rat model** (Fig. 2D, E)**. The quantitative assay of rat whole skin thickness demonstrated that the skin became thicker in the middle of expansion (expansion volume = 100 mL) and was thinner at the end of the expansion, when complications typically occurred (expansion volume = 200 mL)** (Fig. 2F, G).** Detection of the epidermal stem cell biomarkers (keratin 15 [KRT15] and keratin 5 [KRT5]) indicated that these stemness markers change along with the skin tissue thickness after different volumes of mechanical stretch, when skin thickness increased, the expression of stem cell markers increased markedly, and stem cell markers decreased significantly when skin thickness was reduced** (Fig. 2H).** Immunofluorescence staining for proliferation and stemness markers in the rat model and in human skin samples that underwent mechanical stretch revealed similar results **(Fig. 2I).** These results suggest that in the early stage of mechanical stress stimulation, epidermal stem cells proliferate actively, at this time the whole skin layer thickness increased. When the mechanical stress stimulation exceeds the threshold of the skin tissue, the proliferation ability of epidermal stem cells decreases and the skin thickness declines, and the complications (like ulcer, necrosis) are more likely to occur, which is consistent with the clinical observed phenomena.

### Identification of Hub Gene for Mechanical Stretch-induced Skin Regeneration in Rats

**Fig 3:**
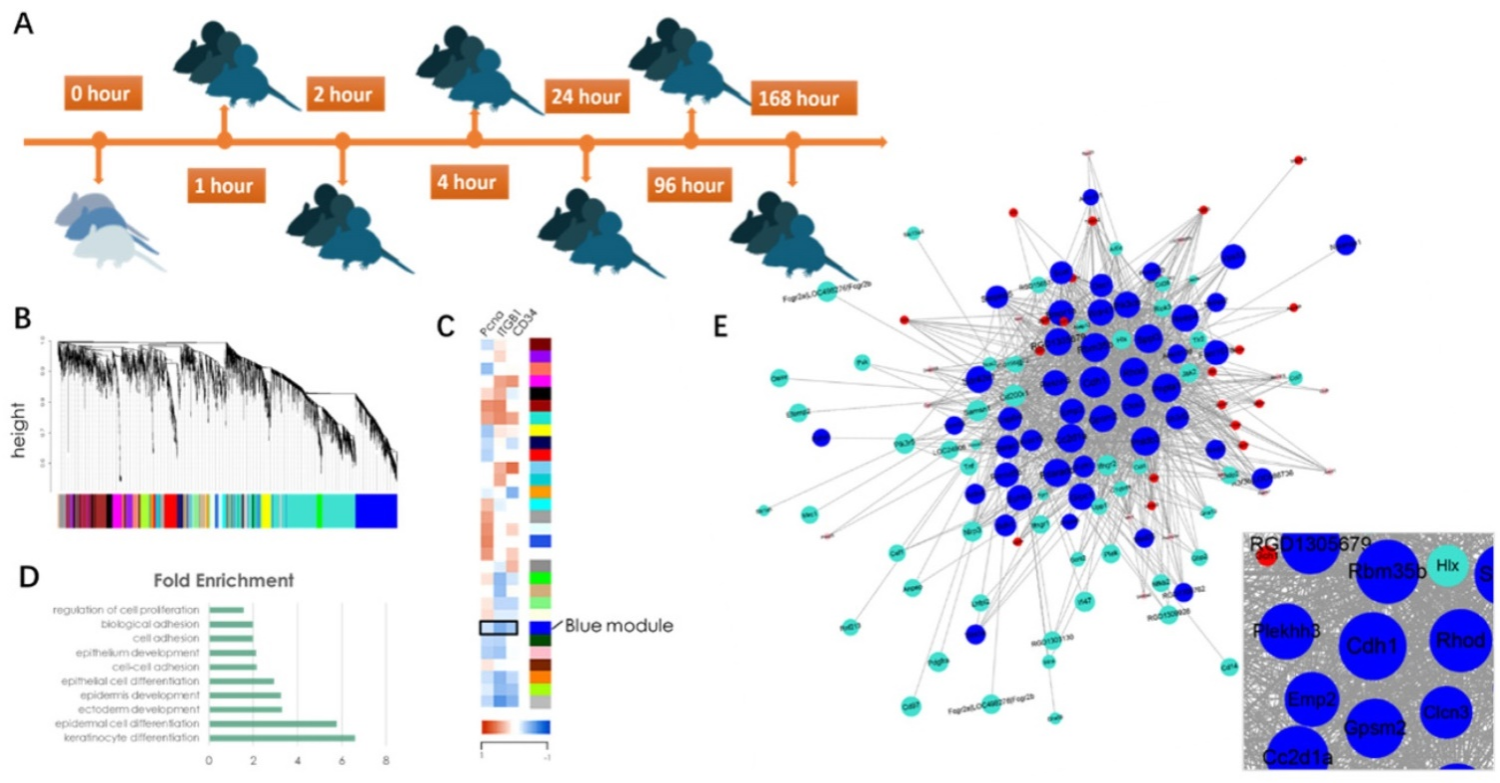
Diagram of identification of significant gene modules and hub genes during mechanical stretch-induced skin regeneration. (*A*) Rat skin tissue was collected at six time points after mechanical loading; RNA isolation was used for microarray analysis. (*B*) Gene module identification. (*C*) Correlation analysis between gene modules and proliferation and stemness molecular markers. (*D*) GO information of the BLUE module. (*E*) Gene coexpression network within the BLUE module and out-module gene coexpression network (red and turquoise module). Genes associated with significant GO terms were analyzed and identified by the gene co-expression network using the k-core algorithm. Nodes represent genes; edges indicate the interaction between the genes. The area of each node represents the k-core value within the module, and the edge correlates with the capacity for modulating adjacent genes.

To reveal the underlying mechanism of mechanical stretch-induced skin regeneration, we conducted a microarray **(**E-MTAB-8304**)** on rat skin tissue samples that had undergone different stages of mechanical loading **(Fig. 3A)**. A gene co-expression network was constructed via WGCNA** (Fig. 3B)** and a total of 30 gene modules were identified. Correlation analysis with epidermal stem cell and proliferation molecular markers (Keratin 15[KRT15], integrin β1 [ITGB1], proliferating cell nuclear antigen [PCNA]) revealed that a BLUE gene module was negatively correlated with these markers, which indicated that the module contains genes that regulate cell proliferation and regeneration negatively** (Fig. 3C)**. To verify this hypothesis, Gene Ontology (GO) information was used to provide deeper insight into the biological process of the BLUE module. The results demonstrated that the BLUE module comprises genes that function as negative regulators of cell proliferation and that regulate cell-cell adhesion and cell differentiation positively** (Fig. 3D)**. The connectivity index (k) was determined for all network genes by taking the sum of their connection strengths (co-expression similarity) with all other genes in the network. The connectivity index represents network “hubs” and is localized. The TOM function 14 was used to calculate the connectivity (k-core value) of every signal gene in the network, genes with higher k-core values (but not fold change) are more centralized in the network and have stronger capacity for modulating adjacent genes. CDH1 was located in the center of the BLUE module **(Fig. 3E)**, its encoded protein is the calcium-dependent cell-cell adhesion protein E-cadherin. Based on the theory, we hypothesized that CDH1 has the strongest capacity for moderating genes in the BLUE module and that interfering with the CDH1 expression level would significantly upregulate the proliferation and regeneration-related biological processes.

### E-Cadherin Plays a Major Role in Mechanical Stretch-induced Keratinocyte Dedifferentiation

**Fig 4:**
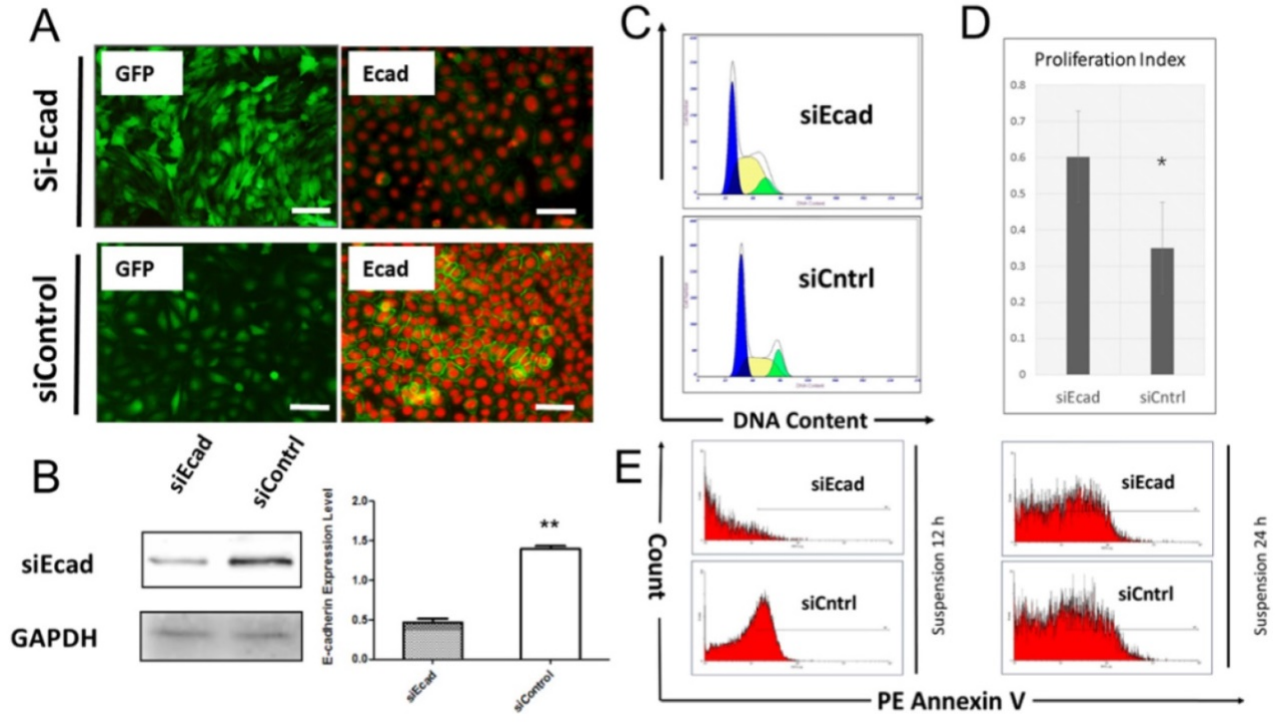
Inhibition of CDH1 expression promotes keratinocyte proliferation and prevents anoikis in vitro. (*A*) (Left column) Immunofluorescence staining of HaCaT cells stably transfected with lentivirus vector (shEcad) or green fluorescent protein (GFP, green); (right column) immunofluorescence staining of human primary keratinocytes transiently transfected with siEcad+HKP complex. E-cadherin was downregulated compared with the siControl group. Cells were stained for E-cadherin (green) and propidium iodide (red). (*B*) Western blotting and quantitative assay of HaCaT cells stably transfected with E-cadherin siRNA. E-cadherin was downregulated in the siEcad group compared with the siControl group (*P* < 0.01). (*C*) Flow cytometry analysis of the cell cycle. The siEcad group had significantly more S-phase cells than the siControl group. (*P* < 0.05). (*D*) The PI was calculated according to flow cytometry data: the siEcad group PI was significantly higher than that of the siControl group. (*E*) Anoikis assay; representative FACS histograms indicate the percentage of apoptotic cells in suspension as determined by annexin V binding. FACS was performed 12 h and 24 h after suspension culture. Values present the means ± SD. *p<0.05, **p<0.01, ***p<0.001.

To verify the hypothesis, RNA interference (RNAi) was used to target CDH1 expression in both the HaCaT cell line and primary human keratinocytes in vitro. The HaCaT cells were infected with lentivirus vector to knock down CDH1 expression levels, and cells were successfully selected in medium containing puromycin (1 μg/mL). The HaCaT cell morphology was significantly changed after puromycin selection, where the cells lost their epidermal cell appearance **(Fig. 4A, left)**. The keratinocytes were transfected by with a short interfering RNA histidine lysine copolymer (siRNA+HKP) cocktail, and cell morphology did not change significantly** (Fig. 4A, right)**. Western blotting demonstrated that HaCaT cells infected with lentivirus vector expressed significantly lower levels of E-cadherin, and quantitative assay verified the results** (Fig. 4B)**. The transfected HaCaT cell line was used for flow cytometric cell cycle analysis. **Figure 4C** shows more S-phase cells (yellow area) among HaCaT cells transfected with E-cadherin siRNA (siEcad) compared to cells transfected with control siRNA (siControl), which indicated more rapid cell proliferation. Furthermore, the siEcad group had a significantly higher proliferation index (PI) than the siControl group** (Fig. 4D)**. Next, the stable transfected HaCaT cells underwent anoikis assay. Figure 4E shows that after 12-h suspension culture, siEcad cells were protected from anoikis compared with the siControl cells; after 24-h suspension culture, there was no significant different between the two groups.

**Fig 5:**
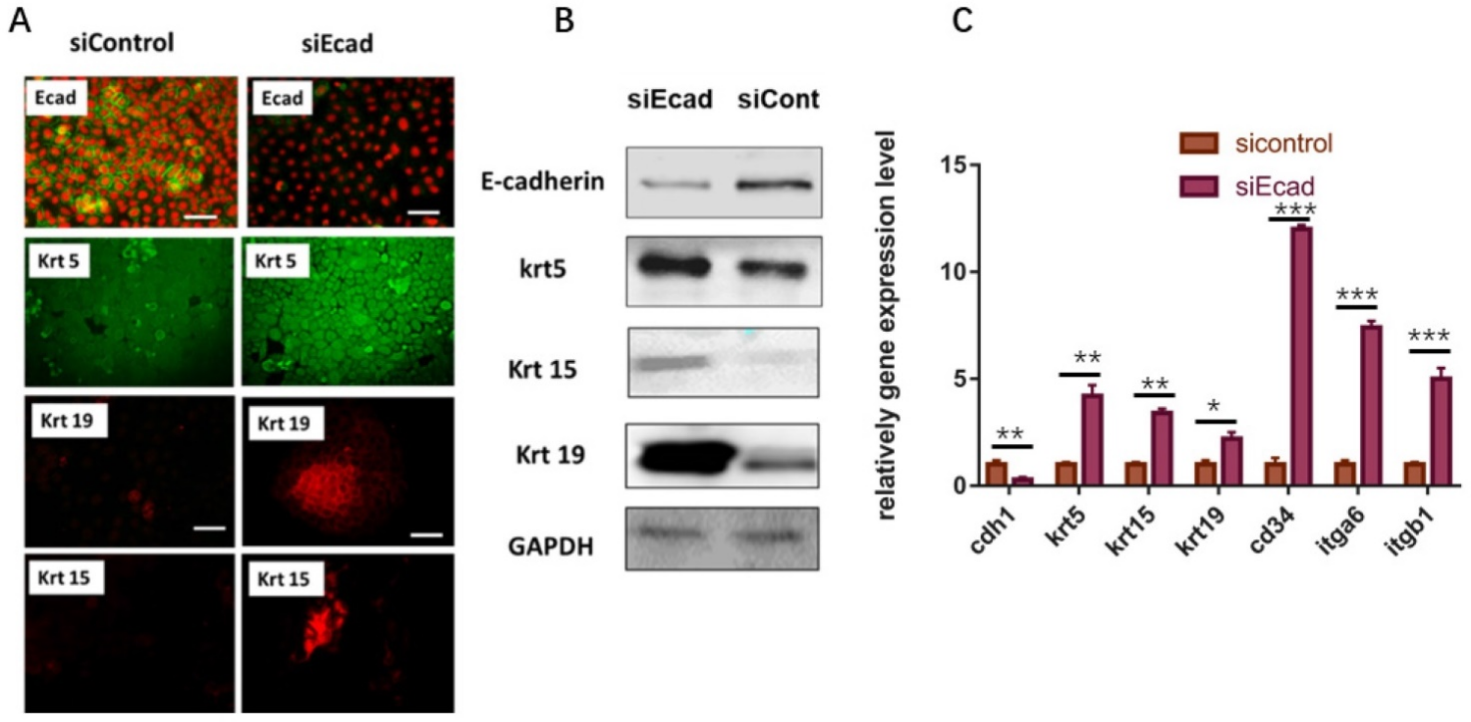
Human primary keratinocytes gain stemness after transient transfection with siEcad+HKP complex. (*A*) Human primary keratinocytes underwent dedifferentiation after siEcad+HKP treatment. The cells were stained for E-cadherin (green), Propidium Iodide (red), KRT5 (green), KRT19 (red), and KRT15 (red). N = 3-5 rats per condition over four experiments. Scale bar: 50 µm. (*B*) Western blotting demonstrated that, after siEcad+HKP treatment, human primary keratinocytes expressed higher levels of KRT5, KRT15, and KRT19, but lower levels of E-cadherin. *N* = 3-5 experiments. (*C*) RT-PCR results for siEcad+HKP-treated human primary keratinocytes. CDH1 mRNA expression was reduced after the treatment, and KRT5, KRT15, KRT19, CD34, ITGB1, and ITGA6 mRNA expression increased significantly. N = 3 experiments. Values present the means ± SD. *P<0.05 **P<0.01, **P<0.01. ***P < 0.001.

Subsequently, the stemness of human primary keratinocytes was detected. As the HaCaT cell line is an immortalized cell line, the initial expression level of epidermal stem cell markers is relatively high, so we opted to use primary human keratinocytes to examine the stem cell markers after CDH1 RNAi. After 36-h transient transfection of siEcad+HKP complex, the primary human keratinocytes gained more stemness, where the mRNA expression level of several epidermal stem cell markers (KRT5, KRT15, KRT19, CD34, ITGB1, ITGA6) was significantly elevated compared to that of the siControl+HKP group and blank control group** (Fig. 5C)**. Immunofluorescence staining demonstrated increased expression of hair follicle stem cell markers (KRT15 and KRT19) after siEcad transfection** (Fig. 5A);** western blotting verified the immunofluorescence staining results** (Fig. 5B)**. These results indicate that the human primary keratinocytes underwent dedifferentiation after siEcad+HKP treatment.

We also used monoclonal anti-E-cadherin antibodies to suppress its function in HaCaT cells during mechanical loading (Flexcell system). The results demonstrated that using antibody could significantly enhance the expression level of stemness markers in HaCaT cell line. The stemness markers were significantly higher in E-cadherin antibody group with mechanical loading compared to mechanical loading group (supplementary figure 2).

### E-Cadherin Knockdown Induced Keratinocyte Dedifferentiation in vivo

**Fig 6:**
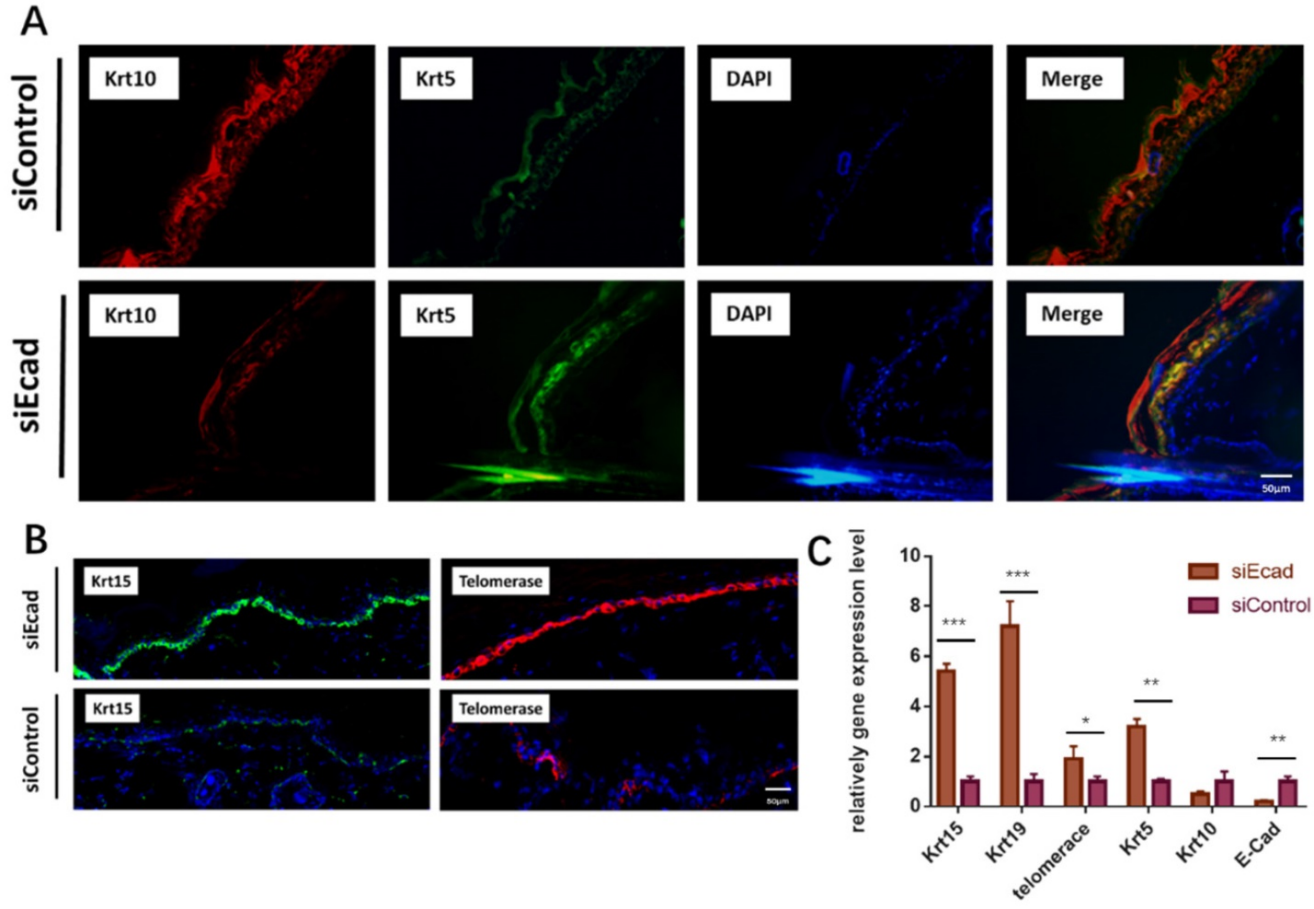
E-cadherin knockdown induces keratinocyte dedifferentiation in vivo. (*A*) E-cadherin knockdown induced epidermal dedifferentiation. KRT5 expression (green) increased while KRT10 expression (red) was decreased in the epidermis; blue, DAPI. Scale bar: 50 µm. *N* = 3 experiments. Scale bar: 50 µm. (B) E-cadherin knockdown induced enrichment of KRT15+ epidermal cells (green) and activation of telomerase (red); DAPI (blue). Scale bar: 50 µm. *N* = 3 experiments. (*C*) PCR showed increased expression of the epidermal stem cell markers KRT15, KRT19, KRT10, KRT5, and telomerase after siEcad+HKP treatment. *N* = 3 experiments. *P < 0.05, **P < 0.01, ***P < 0.001.

To ascertain if siEcad plays the same role in vivo, we injected siEcad+HKP complex (2 µg/mL) four times per week into the skin of Lewis rats. Fourteen days after siEcad+HKP complex injection, the treated group exhibited signs of dedifferentiation. KRT5 is an undifferentiated marker of epidermal stem cells that is normally expressed in the basal layer of the skin, and KRT10 is located in differentiated keratinocytes under normal conditions. After siEcad+HKP treatment, KRT5 expression was increased while KRT10 expression in the epidermis was decreased, suggesting that CDH1 knockdown can induce keratinocyte dedifferentiation **(Fig. 6A)**. KRT15 is a hair follicle stem cell marker usually expressed within epidermal basal layer and hair follicle stem cell niche, and KRT15+ epidermal cells participate in the repair of wounded skin tissue. siEcad+HKP-treated cells were more likely to express KRT15 in the basal layer than siControl cells** (Fig. 6B, left)**, thereby promoting epidermal regeneration. Telomerase (TERC/Terc), also called terminal transferase, is active in normal stem cells and most cancer cells, but is normally absent or found at very low levels in most somatic cells. The immunofluorescence staining demonstrated that telomerase activity increased after siEcad+HKP treatment. **Figure 6C** shows the PCR results for the changes in the stem cell and differentiation markers after siEcad+HKP interference, where KRT15, KRT19, KRT5, and telomerase were significantly increased, and KRT10 was decreased. Taken together, these data suggest that siEcad can promote skin regeneration via epidermal cell dedifferentiation.

We also checked the function of monoclonal anti-E-cadherin antibodies in vivo. The results were consistent with the in vitro group. The stemness markers (KRT15, KRT19, KRT5) were significantly higher in E-cadherin antibody group + expansion group compared to expansion group (supplementary figure 2).

### E-Cadherin Knockdown Induced dermal collagen deposition

**Fig 7:**
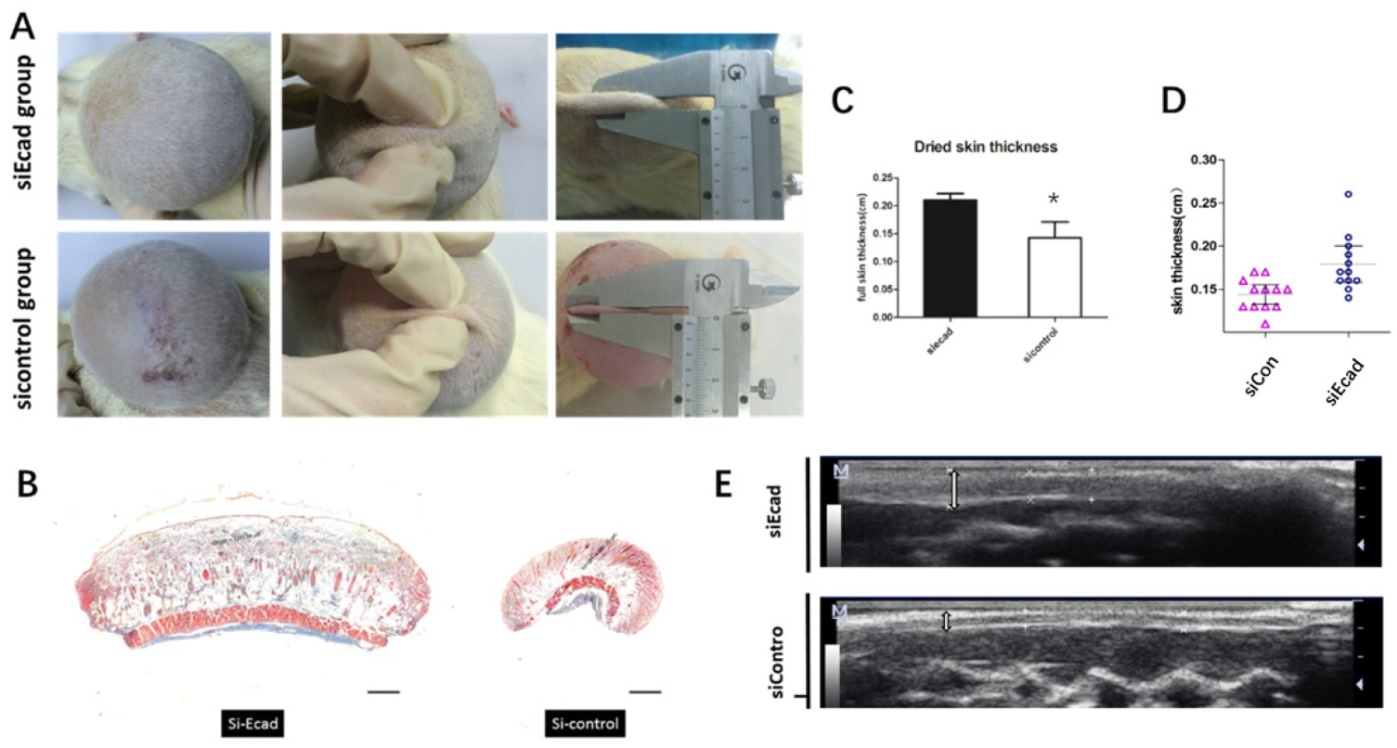
The effect of E-cadherin knockdown on mechanically stretched skin. (*A*) Significantly thicker skin tissue was observed in the siEcad+HKP group; 60% (3/5) of the siControl group developed complications (e.g., flap necrosis); the siEcad+HKP group developed no complications. (*B*) Masson trichrome staining showing more intact flap structure, increased collagen production, and better organized ECM in the siEcad+HKP group compared to the siControl group. Scale bar: 100 mm. *N* = 3-5 rats over three experiments. (*C*) Dried skin thickness (formalin dehydration) demonstrating that the siEcad+HKP group had significantly thicker skin than that the siControl group. *P < 0.05. *N* = 3 rats/group repeated 3-5 experiments. (*D*) ultrasound determined skin thickness of siEcad on day 14. The siEcad+Accell group showed significantly increased flap thickness compared with the siControl group. *N* = 3 rats/group repeat 3-5 experiments. (*E*) Ultrasound images of the siEcad+HKP group and siControl group on day 14. The siEcad+HKP group showed increased flap thickness compared with the siControl group.

Fourteen days after siEcad+HKP complex injection, the treated group exhibited significantly thicker skin than the siControl group **(Fig. 7A)**; therefore, complications (such as necrosis) were prevented. After 200-mL skin expansion, flap necrosis occurred in the siControl group (3/5 rats), and the skin tissue thickness was reduced, while the siEcad+HKP group had thickened skin, no complication was observed. Masson trichrome staining showed that, on day 14, the siEcad+HKP group had significantly increased flap thickness compared with the siControl group **(Fig. 7B-E)**. Histological staining of the stretched flap showed an increase in total thickness (especially dermal thickness).

**Fig 8:**
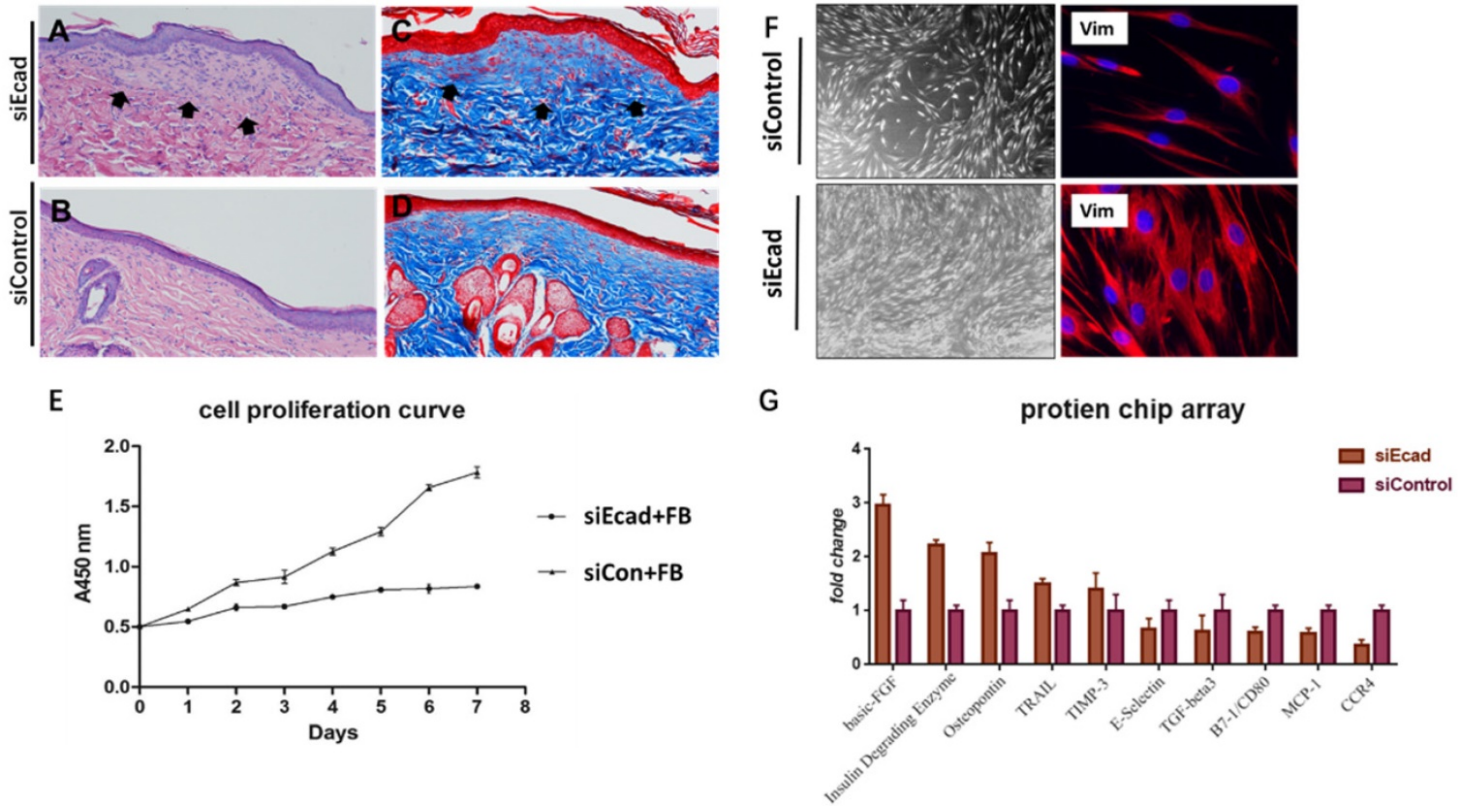
E-cadherin knockdown induces dermal collagen deposition in rat skin. (*A*) Hematoxylin-eosin (HE) staining of rat skin after mechanical loading and siEcad+HKP treatment. Arrowhead shows newly formed collagen deposited under the epidermis layer. (*B*) HE staining of rat skin after mechanical loading and siControl treatment. (*C*) Masson trichrome staining of rat skin after mechanical loading and siEcad+HKP treatment. Arrowhead shows newly formed collagen deposited under the epidermis layer. (*D*) Masson trichrome staining of rat skin after mechanical loading and siControl treatment. (*E*) Cell proliferation curve of fibroblasts cultured with siEcad HaCaT and siControl HaCaT culture fluid. (*F*) Immunofluorescence staining of vimentin (red) and DAPI (blue) showing the morphology of fibroblasts cultured with siEcad HaCaT and siControl HaCaT culture fluid on day 3. (*G*) Protein chip array results of the effect of siEcad HaCaT exocrine cytokine on fibroblast proliferation and collagen deposition.

E-cadherin is mainly expressed in epidermal differentiated epidermal cells, however, interference its expression eventually caused dermis thickening. Masson trichrome staining also showed a more intact flap structure, increased collagen production, and better organized extracellular matrix (ECM) in the siEcad+HKP group. New collagen formation was found beneath the epidermis in the siEcad+HKP group **(Fig. 8A-D)**, which indicated that the interference of epidermal cells affected dermal fibroblast collagen deposition. To investigate the underlying cause, the culture fluid of HaCaT cells permanently transfected with lentivirus vector (siEcad and siControl) was used to culture human primary fibroblasts, and Cell Counting Kit-8(CCK8) was used to observe its influence on the cell proliferation curve **(Fig. 8E)**. A significantly rapid proliferation rate was detected in fibroblasts cultured with the siEcad HaCaT culture fluid, and they exhibited larger cell sizes with abundant cytoplasm** (Fig. 8F)**. These outcomes imply that, after E-cadherin knockdown, epidermal cells may influence dermal fibroblasts via exocrine secretions. Accordingly, protein chip array was used to detect the cytokines produced by siEcad HaCaT cells **(Fig. 8G)**, the collagen formation-related cytokines, such as basic fibroblast growth factor (bFGF) and secreted phosphoprotein 1 (SPP1) were significantly elevated after treatment, while E-selectin, transforming growth factor β3 (TGFB3), and CD80 were significantly decreased.

### E-Cadherin Knockdown Induced Keratinocyte Dedifferentiation through the β-Catenin-FOXO1-KLF4 Pathway

**Fig 9:**
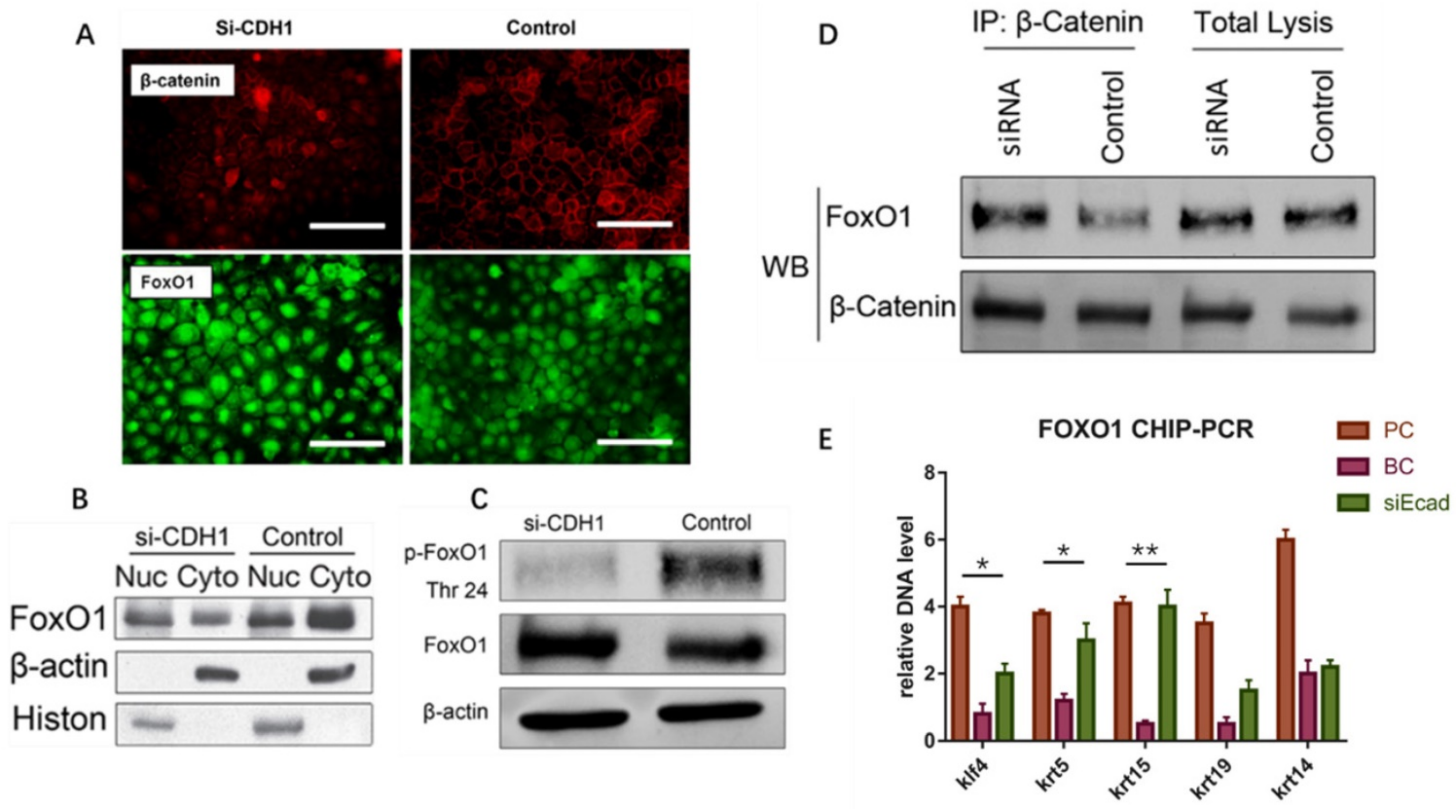
E-cadherin knockdown induces keratinocyte dedifferentiation through the β-catenin-FOXO1-KLF4 pathway. (*A*) E-cadherin knockdown promoted β-catenin and FOXO1 nuclear localization. Image shows immunofluorescence staining of β-catenin (red) and FOXO1 (green). (*B*) Western blot of FOXO1 nuclear localization signal after E-cadherin knockdown. (*C*) Western blot of FOXO1 dephosphorylation state after E-cadherin knockdown. (*D*) Immunoprecipitation demonstrating β-catenin and FOXO1 interaction after E-cadherin knockdown. (*E*) FOXO1 ChIP-PCR showing significantly higher relative DNA levels of KLF4, KRT5, and KRT15 after E-cadherin knockdown as compared to the control group. *N* = 3 experiments. *P < 0.05, **P < 0.01.

**Fig 10:**
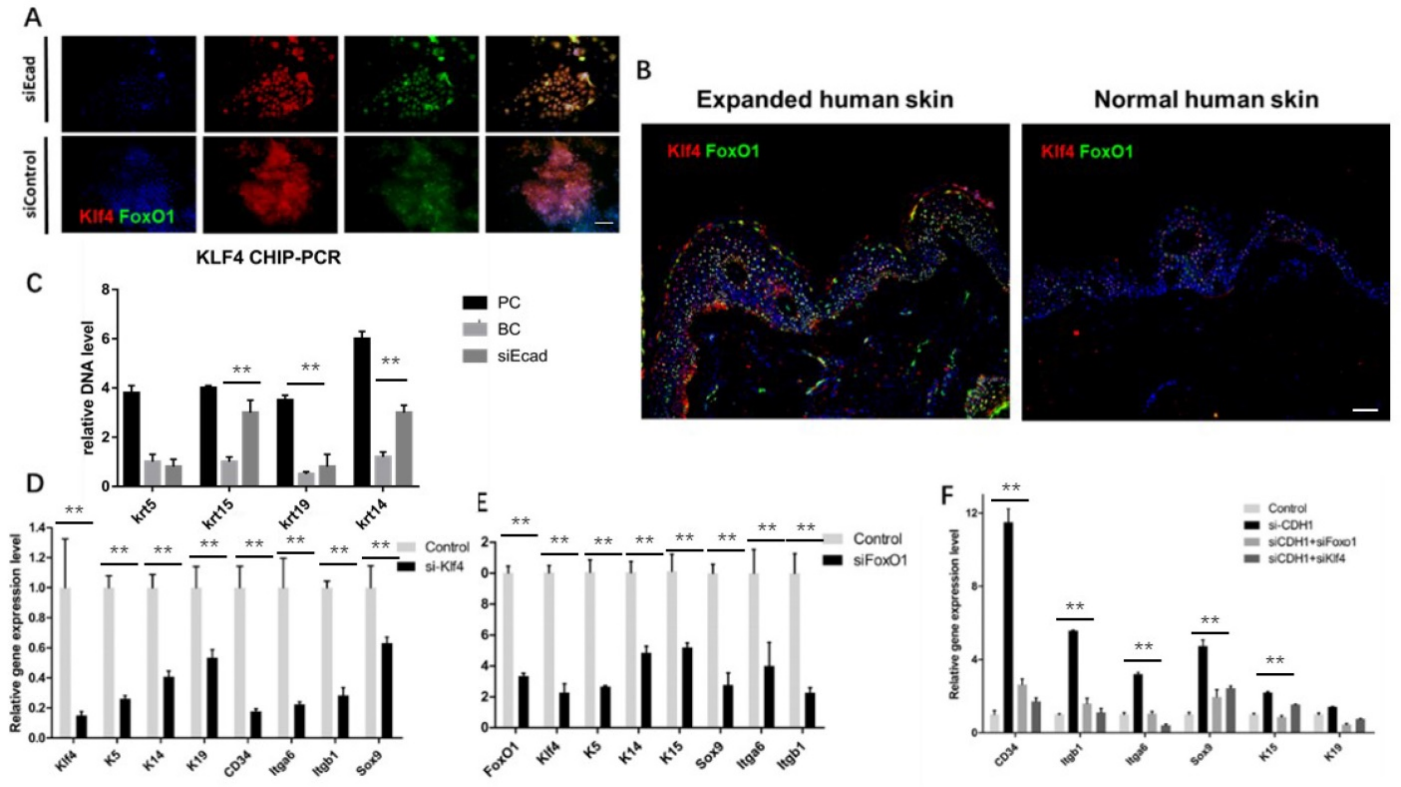
Effect of KLF4 and FOXO1 on stemness markers in human keratinocytes. (*A*) Immunofluorescence staining of KLF4 and FOXO1 showing that E-cadherin knockdown promoted their nuclear localization. (*B*) Immunofluorescence staining of KLF4 and FOXO1 in human skin tissue before and after mechanical loading. Stretched skin showed significantly more KLF4 and FOXO1 nuclear localization than normal skin. (*C*) *KLF4* ChIP-PCR indicating significantly higher relative DNA levels of KRT14, KRT19, and KRT15 after E-cadherin knockdown as compared to the control group. *N* = 3 experiments. **P < 0.01. (*D*) qPCR of siKLF4 keratinocytes: stemness markers were repressed after interference.* N* = 3 experiments. **P < 0.01. (*E*) qPCR of siFOXO1 keratinocytes: stemness markers were repressed after interference. *N* = 3 experiments. **P < 0.01. (*F*) qPCR of CDH1/KLF4 or CDH1/FOXO1 keratinocytes: stemness markers were repressed after interference; CDH1/KLF4 or CDH1/FOXO1 double interference diminished the dedifferentiation caused by *CDH1* knockdown. *N* = 3 experiments.

The E-cadherin/β-catenin complex plays an important role in maintaining epithelial integrity, and interference of E-cadherin expression promotes β-catenin nuclear localization, activating the Wnt signaling pathway, as reported in immortalized human breast epithelial cells (HMLE) 15. We verified this in human keratinocytes **(Fig. 9A)**. To confirm whether Wnt signaling pathway activation leads to keratinocyte dedifferentiation, Dickkopf (DKK) proteins(StemRD, USA), secreted proteins that inhibit Wnt signaling by directly binding to LDL receptor-related protein (LRP)5/6 16, were applied. However, the Wnt inhibitor could not reverse the dedifferentiation induced by E-cadherin loss (Supplementary File, figure 1), implying that another signaling pathway regulates keratinocyte fate. Intriguingly, FOXO1, a TF that plays important roles in regulating gluconeogenesis and glycogenolysis, began to localize to the nucleus when E-cadherin expression was knocked down **(Fig. 9A)**. Before E-cadherin was knocked down, it mainly expresses in cytoplasm. Western blotting verified the dephosphorylation and nuclear localization of FOXO1 after E-cadherin knockdown **(Fig. 9B, C)**. Immunoprecipitation was conducted to determine whether β-catenin and FOXO1 interacted in the nucleus, and verified their interaction after interference of E-cadherin expression** (Fig. 9D)**. FOXO1 is an essential regulator of pluripotency in human embryonic stem cells 17. To address the potential function of FOXO1 in human keratinocytes, chromatin immunoprecipitation (ChIP)-PCR was conducted to reveal its downstream target genes. We found that FOXO1 regulated epidermal stem cell markers such as KRT5 and KRT15, but that FOXO1 does not directly transcribe KRT19 and KRT14. However, knockdown of FOXO1 expression levels (via siFOXO1+HKP complex treatment) affected many more epidermal stem cell markers **(Fig. 10E)**, which indicates the existence of other stemness regulation pathways for FOXO1. Since KLF4 is a target gene of FOXO1 in B cells 18 and known as an induced pluripotent stem (IPS) cell induction factor, we investigated whether FOXO1 regulates KLF4 in human keratinocytes. The ChIP-PCR was used to verified this hypothesis **(Fig. 9E)**, and the results shows that KLF4 is a FOXO1 target gene in keratinocytes.

Immunofluorescence staining showed that E-cadherin knockdown promoted KLF4 and FOXO1 nuclear localization in human primary keratinocytes** (Fig. 10A)**. Furthermore, human skin tissue demonstrated significantly more KLF4 and FOXO1 nuclear localization in the epidermal layer than normal human skin tissue did after mechanical loading **(Fig. 10B)**, implying that both KLF4 and FOXO1 play roles in CDH1/mechanical loading-induced epidermal cell dedifferentiation. To address the role of KLF4 and FOXO1 in human keratinocyte dedifferentiation, KLF4 ChIP-PCR was used to detect the KLF4 downstream target genes. KRT14, KRT15, and KRT19, but not KRT5, were directly transcribed by KLF4. KLF4 and FOXO1 knockdown significantly downregulated a variety of epidermal stem cell markers **(Fig. 10D, E)**, and CDH1/KLF4 or CDH1/FOXO1 double interference diminished the dedifferentiation caused by CDH1 knockdown **(Fig. 10F)**. However, the two TFs did not transcribe all of these stem markers, other E-cadherin downstream pathways should be explored to clarify keratinocyte dedifferentiation.

## Discussion

### CDH1 is the critical factor in mechanical stretch induced skin regeneration

Mechanical strain regulates the development, organization, and function of multicellular tissues, but mechanisms linking mechanical strain and cell-cell junction proteins to cellular responses are poorly understood 19. Clinically, mechanical force is widely used to regenerate skin for post-burn or trauma repair, but its underlying mechanism remains unknown. Hence, in this study, we first reveal that mechanical stretch induces skin regeneration through keratinocyte dedifferentiation, and E-cadherin downregulation initiates the process. Further, we found that E-cadherin downregulation can induce keratinocyte dedifferentiation through the β-catenin-FOXO1-KLF4 pathway.

Through the WGCNA bioinformatics method, we found that CDH1 is the hub gene of mechanical stress-induced skin regeneration. The classic cadherins couple with neighboring cells through *trans* interactions between opposing extracellular domains and force-dependent linkage of the cytoplasmic domain to the actin cytoskeleton through β-catenin and α-catenin 20-22. The association of E-cadherin with mechanical stress-induced cell cycle entry via activation of the downstream transcriptional activators Yes-associated protein 1 (YAP1) and β-catenin has been reported 23, indicating that E-cadherin should be a crucial trigger for the downstream proliferation signaling cascade.

### Skin alteration after E-cadherin loss

E-cadherin is thought to mediate intercellular adhesion in the mammalian epidermis and in hair follicles as the adhesive component of adhesion junctions. The phenotype of E-cadherin ablation in the epidermis and hair in both mutant mice and Cre/*loxP* mice has been reported 24, 25. Tinkle et al.^25^ reported that E-cadherin loss may be incompatible with epithelial tissue survival, whereas partial compensation can result in alterations in differentiation and proliferation. On the other hand, Young et al. (24) reported that the loss of E-cadherin in keratinocytes led to the loss of adherens junctions and altered epidermal differentiation. Both studies reported loss of hair coat after E-cadherin loss. Here, we found that mechanical stress could induce keratinocyte dedifferentiation. Since CDH1 is the hub gene moderating the regeneration-related gene module, CDH1 interference also led to altered epidermal differentiation, which is consistent with the previously published literature. However, we did not find distinct hair coat loss after CDH1 interference, suggesting that hair loss requires a greater degree of CDH1 decrease. In addition, we found that the CDH1 interference leads to dermal thickening and significant collagen deposition. Therefore, an epidermal cell supernatant protein chip was used to detect the change in epidermal secretion after E-cadherin loss. Several collagen formation-related cytokines, such as bFGF and SPP1 were significantly elevated after treatment, while E-selectin, TGFB3, and CD80 were significantly decreased after siEcad interference. These results suggest that the dermal thickening after CDH1 knockdown is due to altered exocrine secretion after epidermal loss of E-cadherin and explain why mechanical stress-induced changes to CDH1 expression can affect the state of the entire skin layer extensively.

### FOXO1 function in CDH1 knockdown

FOXO proteins are essential regulators of the pluripotency of multiple stem cell lines 17, 26-28 and are required for the maintenance of cancer stem cells 29, 30. As FOXO1 interacts with E-cadherin/β-catenin, promoting the downstream signaling pathway, we investigated whether FOXO1 contributes to the stemness alteration after E-cadherin/ CDH1 interference. We found that FOXO1 activation is the major factor in CDH1 knockdown-induced keratinocyte dedifferentiation and that blocking *FOXO1* expression could reverse the CDH1 knockdown-induced keratinocyte dedifferentiation. Increased FOXO1 and KLF4 nuclear accumulation was found in both the stretched skin tissue and CDH1-interfered keratinocytes, and ChIP-PCR indicated that the activation of the two TFs stimulated the expression of the stemness-related markers. Zhang et al. (17) reported that FOXO1 depletion was accompanied by rapid downregulation of pluripotent OCT4 (POU class 5 homeobox 1), NANOG (Nanog homeobox), and SOX2 (SRY-box 2) expression in FOXO1 short hairpin RNA (shRNA)-treated mice embryonic stem cells. These alterations were concomitant with a significant loss of pluripotency surface markers.

**Fig 11:**
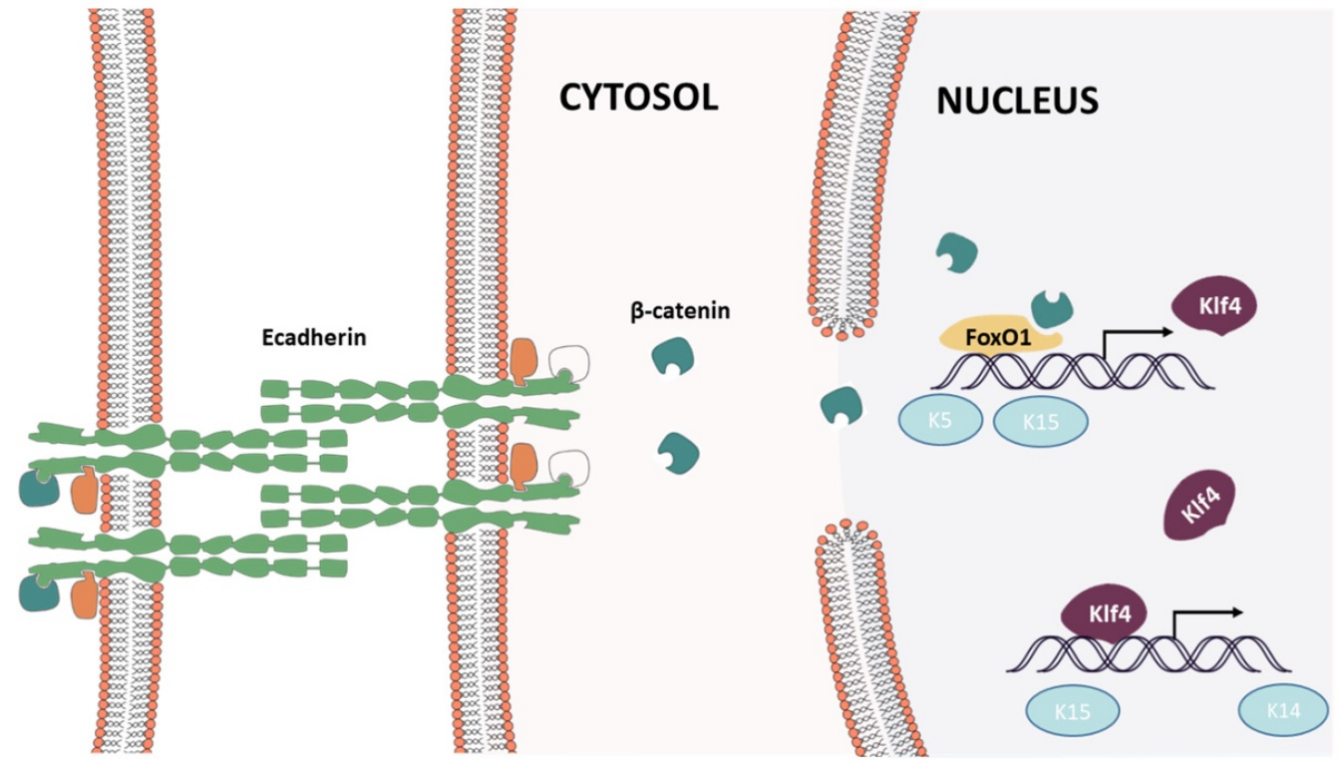
Epidermal cell intracellular signal transduction after mechanical loading

The present study demonstrates that mechanical stretching can induce keratinocyte dedifferentiation by downregulating E-cadherin and that knockdown of CDH1 alone has a similar effect on keratinocytes and promotes β-catenin nuclear localization, activating the Wnt signaling pathway. β-Catenin combined with FOXO1 and directly regulated pluripotency gene expression (KLF4, KRT5, KRT15). Thus, FOXO1 emerged as a critical regulator in E-cadherin-mediated keratinocyte dedifferentiation, and FOXO1 knockdown reversed the dedifferentiation caused by CDH1 downregulation **(Fig. 11)**. KLF4, a downstream gene of FOXO1, also regulates pluripotency gene expression (KRT14, KRT19, KRT15). Together, these results indicate that CDH1 is a component of the human keratinocyte pluripotency circuitry and that the FOXO1-KLF4 pathway is recognized a key regulator thereof. These findings have critical implications for stem cell biology, development, longevity, and reprogramming.

## Materials and Methods

### Skin Stretching Model

We followed the Guide for the Care and Use of Laboratory Animals (National Institutes of Health, Bethesda, MD, USA) in all animal procedures. The Committee on the Ethics of Animal Experiments of Shanghai Jiao Tong University School of Medicine (Shanghai, China) approved the study protocol.

Twenty-one male Lewis rats (6 weeks old) were allocated randomly to the stretching group (*N* = 18) and control group (*N* = 3). Rats in the stretching group were assigned randomly to six time points (*N* = 3 PER time point): 1, 2, 4, 24, 96, and 168 h. The rats were anesthetized (5% chloral hydrate, 250 mg/kg body weight) and 10-mL silicon expanders (Shanghai Xin Sheng Biomedical, Shanghai, China) were implanted subcutaneously on the dorsal sides. In the stretching group, 0.9% saline was injected through the port of the tissue expander to maintain 60 mmHg intracapsular pressure, while nothing was injected in the control group and the expander was kept uninflated. At each time point, full-thickness skin specimens from the stretched skin area were collected from the stretching group. From the control group, full-thickness skin specimens from the implanted area were collected at 168 h (7 days). The animals were euthanized immediately after sample collection.

### Microarrays

Total RNA was extracted from skin specimens using TRIzol (Invitrogen, Carlsbad, CA, USA) and purified using an RNeasy Mini Kit (Qiagen, Valencia, CA, USA). RNA was qualified by absorbance values of 1.8-2.0 at 260 nm and 280 nm, and clear bands after polyacrylamide gel electrophoresis. RNA samples from rat skin specimens were hybridized, washed, and scanned with GeneChip Rat Exon 1.0 ST Arrays according to the manufacturer's instructions (Affymetrix, Santa Clara, CA, USA). Gene expression was normalized with a robust multi-array average in the dataset. Differentially expressed genes were determined by one-way analysis of variance (ANOVA, *P* < 0.05). All data generated were compliant with the minimum information about a microarray experiment (MIAME) standard 31.

### WGCNA for Hub Gene Detection

WGCNA was performed using the WGCNA package in R (R Foundation, Vienna, Austria) 32, and only differentially expressed genes were inputted to minimize noise. First, the pairwise correlation matrix was computed for each set of genes. Then, the adjacency matrix was constructed with parameter β determined by the pickSoftThreshold function to fit the scale-free criterion 13, 33. Next, we calculated the topologic overlap matrix (which measures the interconnectedness of the coexpression network). Then, the topologic overlap-based dissimilarity was used as an input for unsupervised hierarchical clustering by employing a dynamic tree-cutting algorithm 34. Branches of the clustering tree were defined as modules, which included a group of coexpression genes and which were summarized by module eigengenes 32, 35. We calculated correlation coefficients between module eigengenes, and merged highly similar modules. After identifying the coexpression modules, we associated these modules with stemness biomarkers, and then identified the most related module. Hub genes (which have a central role in the scale-free network) were located in the most regeneration-related module using a method described previously 36. A hub gene network was plotted with Cytoscape 37 to visualize the entire weighed gene coexpression network.

### Cell Culture

Primary rat keratinocytes were harvested from the dorsal skin of 3-day-old Sprague Dawley rats and cultured with Keratinocyte Medium (ScienCell, Carlsbad, CA, USA) as described previously 38. The HaCaT human keratinocyte cell line, an immortalized spontaneous nontransformed epithelial cell line, was purchased from Cell Lines Service (DKFZ, Eppelheim, Germany). The cells were grown in Dulbecco's modified Eagle's medium (DMEM), high glucose, supplemented with 2 mM L-glutamine, 100 U/mL penicillin/100 mg/mL streptomycin, and 10% fetal bovine serum (FBS). All cell culture reagents were from Life Technologies (Thermo Fisher Scientific, Waltham, MA, USA). The cells were cultured in 75-cm^2^ cell culture flasks at 37°C in a 5% CO_2_ atmosphere. The cells received fresh medium every 2 days and were subcultured every 5 days.

### Anoikis Assay

CDH1 siRNA-treated rat primary keratinocytes were seeded onto 6-well ultra-low attachment plates. After 24 h, the cells were harvested and incubated at 37°C with 0.25% trypsin for 5 min to prevent cell aggregation. Viable cells were counted using trypan blue. For fluorescence-activated cell sorting (FACS), we used the ApoAlert Annexin V-FITC (fluorescein isothiocyanate) Apoptosis Kit (Takara Biotechnology, Shiga, Japan) according to the manufacturer's instructions, and a FACSCalibur flow cytometer (Becton Dickinson, USA).

### Mechanical stress stimulation of keratinocyte

Human primary keratinocyte was transferred to a Biofex 6-well plate (Flexcell, Burlington, NC, USA) and allowed to adhere to the membrane until they reached 90% confluence. The cells were synchronized in the G0 stage by culturing in Keratinocyte Medium (ScienCell, Carlsbad, CA, USA) and then were exposed for 3h to stretch stimulation. Cells without stretching served as controls. Cells were subjected to sinusoidal stretching at 10% strain at a 1.25 Hz frequency. Experiments were performed at 37 °C in 5% CO_2_. RNA samples from cell samples were hybridized, washed, and scanned with GeneChip human Exon 1.0 ST Arrays according to the manufacturer's instructions (Affymetrix, Santa Clara, CA, USA). Gene expression was normalized with a robust multi-array average in the dataset.

### Collection of Human Skin Samples and Histology

Specimens of stretched skin and adjacent normal skin were obtained from patients who had undergone reconstructive surgery using a skin stretching method. Written informed consent for sample collection was obtained from the patients, and the Ethics Committee of Shanghai Ninth People's Hospital (Shanghai, China) approved the sample collection. For histopathology, human and rat skin tissue was fixed with 4% paraformaldehyde, paraffin-embedded, and sectioned (thickness = 5 µm). Masson trichrome staining was performed according to the manufacturer's instructions (Sigma-Aldrich, Saint Louis, MO, USA).

### Immunofluorescence

Primary rat keratinocytes and HaCaT cells were plated in an 8-well confocal chamber (80821; ibidi, Munich, Germany) and treated with siRNA for 48 h. The cells were washed with phosphate-buffered saline (PBS) and fixed in methanol for 10 min. The skin tissue was fixed with 4% paraformaldehyde, paraffin-embedded, and sectioned (thickness = 5 µm). After fixation, the samples were permeabilized and blocked in solution containing 2% bovine serum albumin, 0.5% Tween 20, and PBS (pH 7.3) for 1 h. Antigen retrieval was conducted according to the manufacturer's instructions. The samples were incubated with primary antibody overnight at 4°C and visualized using secondary antibody conjugated with fluorophores that absorbed at 488 nm or 586 nm. Nuclei were stained with 4',6-diamidino-2-phenylindole (DAPI, 1:1000; Sigma-Aldrich) for 10 min and mounted in Dako Mounting Medium (Sigma-Aldrich). The primary antibodies used were antibodies against Ki67 (1:150; Millipore, Bedford, MA, USA), vimentin (1:200; Abcam, Cambridge, UK), E-cadherin (1:200; BD Biosciences), KRT15 (1:100; Abcam), KRT5 (1:200; Abcam), KRT19 (1:100; Abcam), telomerase (1:100; Abcam), KLF4 (1:50, santa cluze Biotechnology, USA), KRT10 (1:100; Abcam), and FOXO1 (1:100, Abcam). Images were acquired using a confocal microscope (Leica, Wetzlar, Germany). Images were analyzed using ImageJ 1.5 (National Institutes of Health, Bethesda, Maryland, USA).

### Quantitative PCR

Total RNA was extracted using TRIzol (Invitrogen); RNA concentrations were determined using a NanoDrop spectrophotometer (ND-8000; Thermo Fisher Scientific). RNA samples were reverse-transcribed into complementary DNA (cDNA) using a Moloney murine leukemia virus reverse transcriptase (M-MLV) system (Promega Biotech, Madison, WI, USA). The primers used are listed in Supplementary Table 1. Quantitative PCR (qPCR) was carried out with SYBR Premix Ex Taq (TaKaRa Biotechnology) on an ABI ViiA 7 system (Applied Biosystems, Foster City, CA, USA). Glyceraldehyde-3-phosphate dehydrogenase (GAPDH) was used as the internal control. The relative mRNA expression was calculated as 2-ΔΔCt (Ct, threshold cycle) and referred to as the fold change in expression in the treated group as compared with the control group 39.

### Western Blotting

Whole-cell protein lysates were prepared with radioimmunoprecipitation (RIPA) buffer containing a protease inhibitor cocktail (Roche, Basel, Switzerland). Membrane, cytoplasmic, and nuclear proteins were extracted using a subcellular protein fractionation kit (78840; Thermo Fisher Scientific) according to the manufacturer's protocols. Then, 40 µg protein lysate (2ug/ul) per sample was loaded onto a 10% sodium dodecyl-polyacrylamide gel and transferred to 0.45-µm polyvinylidene difluoride (PVDF) membranes using a semi-dry transfer system (Trans-Blot Turbo; Bio-Rad Laboratories, Hercules, CA, USA). The membranes were incubated overnight at 4°C with primary antibodies and then incubated with horseradish peroxidase-conjugated secondary antibody (1:4000) for 1 h at room temperature. Protein expression was detected using an electrochemiluminescence kit (Tanon Science & Technology, Shanghai, China), and blot images captured using a chemiluminescence imaging system (6100; Tanon Science & Technology). The primary antibodies used were antibodies against E-cadherin (1:200; BD Biosciences), KRT5 (1:200; Abcam), KRT15 (1:100; Abcam), KRT19 (1:200; Abcam), phosphorylated (p)-FOXO1 (1:1000; Cell Signaling Technology, Beverly, MA, USA), FOXO1 (1:1000; Cell Signaling Technology), β-catenin (1:1000; Cell Signaling Technology), β-actin (1:1000; Cell Signaling Technology), and histone (1:500; Santa Cruz Biotechnology).

### siRNA Transfection

Human and rat CDH1, FOXO1, and KLF4 oligonucleotides were designed using BLOCK-iT RNAi Designer (http://rnaidesigner.thermofisher.com/rnaiexpress) and constructed by GenePharma (Shanghai, China). The sequences were as showed in supplementary table 2.

HKP was provided by the University of Maryland Biopolymer Lab or purchased from AmbioPharm (Shanghai, China), and used for transient transfection according to the manufacturer's instructions with 50 nm CDH1/FOXO1/KLF4 siRNA or scramble siRNA. In vivo intradermal injection of siRNA (100 μg/mL) was performed every 3 days. Six rats each underwent CDH1 siRNA transfection or scramble siRNA treatment. The injections continued for 56 days in total.

### ChIP-qPCR

For ChIP, a 9-cm dish of subconfluent HaCaT cells (10-15 × 10^6^ cells) was fixed with 1% formaldehyde for 10 min at room temperature. The formaldehyde was neutralized by the addition of 125 mM glycine for 5 min. The cells were washed twice in ice-cold PBS and collected by scraping in 1 mL 1% sodium dodecyl sulfate (SDS), 100 mg/L sonicated salmon sperm DNA, and protease inhibitors (15 mg/L aprotinin, 2 mg/L leupeptin, 0.2 g/L phenylmethanesulfonyl fluoride [PMSF]). To eliminate free FOXO1, the lysates were vortexed and insoluble material was collected by centrifugation at 14,000 ×*g* at 4°C for 5 min. The pellets were resuspended in 1 mL 0.25% SDS/200 mM NaCl/100 mg/L sonicated salmon sperm DNA and protease inhibitors and were sonicated to an average fragment size of 1 kb using a microtip on a sonicator (Branson 8150, USA). The remaining insoluble material was removed by centrifugation at 14,000 ×*g* at 4°C for 5 min. The supernatant was diluted with 2 vol 1% Nonidet P-40/350 mM NaCl and incubated for 12 h at 4°C with 10 μL antibody-coated paramagnetic protein G beads (Dynal, Inc. New York, USA)). Each ChIP used 1 μg DO-7 (anti-p53) and 1 μg PEP 2 (anti-Sp1) (Santa Cruz Biotechnology). Immune complexes were collected with a magnet, washed four times in 1% Nonidet P-40/350 mM NaCl/100 mg/L sonicated salmon sperm DNA, resuspended in 125 μL 1% SDS/16 mg/L salmon sperm DNA, and eluted by heating to 85°C for 10 min. Crosslinking was reversed by incubating the eluate for 6 h at 65°C. Samples were diluted with 125 μL water containing 160 mg/L proteinase K, and incubated for 1 h at 50°C. DNA was purified by phenol/chloroform extraction and precipitated with isopropyl alcohol and glycogen.

qPCR was performed on a PE5700 PCR machine using a TaqMan Master Mix (t (Applied Biosystems, USA).). The PCR cycles were 50°C for 2 min to digest dUTP-containing DNA, 95°C for 10 min to activate the Taq Gold polymerase, and then 40 cycles of 95°C for 15 s and 60°C for 1 min, except for the *GAPDH* primers, for which the annealing temperature was 55°C. Total RNA was extracted using TRIzol (Invitrogen), and 2 μg RNA were reverse-transcribed using SuperScript II (Invitrogen). The probe and primer sequences are showed in supplementary table 3.

### DKK protein and E-cadherin antibody treatment

Recombinant human Dkk-1 (20 nM) was added to the Keratinocyte Medium. Plates were incubated for 2 days and q-PCR was conducted to detect the presence of epidermal stem cell markers as described above. 1ug/ml monoclonal E-cadherin antibody (Bio-sciences, San Jose, CA) was applied to block E-cadherin function in vitro and in vivo. Flexcell were incubated for 24 hours and q-PCR was conducted to detect the presence of epidermal stem cell markers as described above. In expansion model, q-PCR was conducted after 2 days after monoclonal E-cadherin antibody injection.

### Statistical Analysis

The western blotting and immunofluorescence results were quantified using ImageJ and analyzed using the *t*-test (two groups) or one-way ANOVA and multiple comparison (≥3 groups) by GraphPad Prism 6 (GraphPad Software, San Diego, CA, USA). Significant differences were defined as P < 0.05.

## Supplementary Material

Supplementary figures and tables.
