# Supplementary material for: CDH1 is Identified as A Therapeutic Target for Skin Regeneration after Mechanical Loading
Source: Int J Biol Sci. 2021 Jan 1;17(1):353–67. doi: 10.7150/ijbs.51309 (PMC7757047; doi:10.7150/ijbs.51309)
Supplement: Supplementary file 1 — Supplementary figures and tables. [file ijbsv17p0353s1.pdf]

Supplementary Material

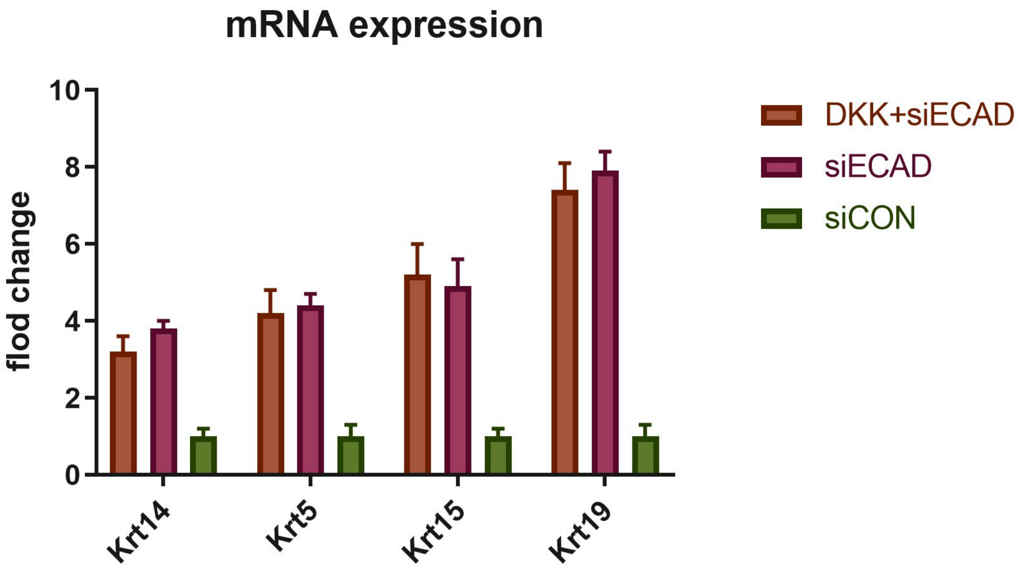

Supplementary Figure1.

Secreted proteins DKK could not reverse the dedifferentiation induced by E-cadherin loss. $N = 3$ experiments.

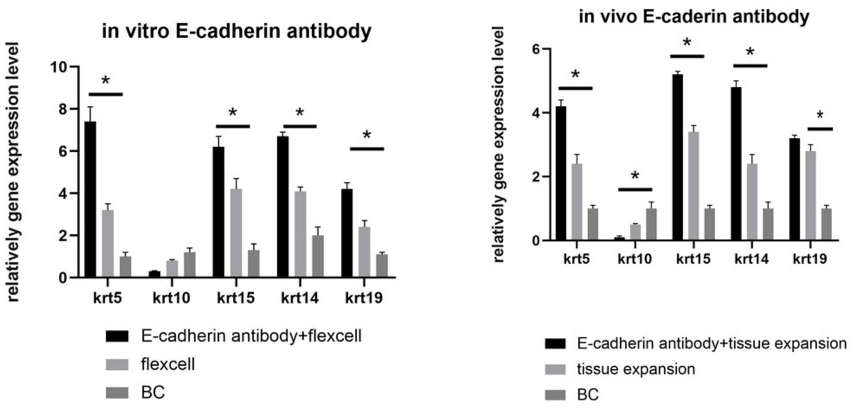

Supplementary Figure2.

In vitro and in vivo results of using E-cadherin antibody to block its function under mechanical loading (in vitro and in vivo). N=3 experiments. *P < 0.05 BC: blank

control

Supplementary table 1. Q-PCR seq

Gene name	Primer sequence
Human Cdh1	5' AAG ACA AAG AAG GCA AGG T 3'
	5' AGA GAG TGT ATG TGG CAA TG 3'
human FOXO1	5' GAG TTA TGG AGG TAT GAG TCA GT 3'
	5' ATT ATG GGG AGG AGA GTC AG 3'
human KLF4	5' CTC GCC TTG CTG ATT GTC TAT T 3'
	5' CAC CTG AAC CCC AAA GTC AAC 3'
human KRT15	5' GGC TCA GAA CCA GGA GTA CAA G 3'
	5' ATT GCT GCT GCT ACC ACC AC 3'
human KRT14	5' ACA GTC CCT ACT TCA AGA CCA TT 3'
	5' GAT GTC GGC TTC CAC ACT C 3'
human KRT5	5' GCC GAG TCC TGG TAT CAG AC 3'
	5' CGC ACT GTT TCT TGA CAT TG 3'
human Krt10	5' AAA TCA AGG AGT GGT ATG AA 3'
	5' GTT GGC ATT ATC AGT TGT TAG 3'
human TERT	5' TGC TCA GGT CTT TCT TTT ATG T 3'
	5' ACC CTC TTC AAG TGC TGT CT 3'
human CD34	5' ATC TCC CAC TAA ACC CTA TAC A 3'

	5' CAC TTC TCT GAT GCC TGA AC 3'
human ITGA6	5' CGT CAG AAA GCA AGG AAG AT 3'
	5' ATA ACA CCG CCC AAA GAT G 3'
human ITGB1	5' GAA GAC TAT CCC ATT GAC CTC TA 3'
	5' TCC GAA GTA ATC CTC CTC AT 3'
human SOX9	5' GAT GAA ATC TGT TCT GGG AAT GT 3'
	5' AAC TGC TGG TGT TCT GAG AGG 3'
human KRT19	5' GCG ACT ACA GCC ACT ACT ACA C 3'
	5' GCC TGT TCC GTC TCA AAC T 3'
human b-actin	5' AAG GTG ACA GCA GTC GGT T 3'
	5' TGT GTG GAC TTG GGA GAG G 3'
rat CDH1	5' CCA ACA GGG ACA AAG AGA CAA 3'
	5' CAA TGA TGA AAA CGC CAA CA 3'
rat Foxo1	5' ACC CCA GTG AAG ACA CCT TTA C 3'
	5' GCT GCT CAC AGA GGA GTA GTT G 3'
rat Klf4	5' GAC CAC CTT GTA TGC TCT TT 3'
	5' CCA TCG TTT AGG CTA TTA GAA 3'

rat Krt15	5' CTC TGG CAA TGA GAA GGT GA 3'
	5' TGG TCT TGA AGT AAT GGC TGT AGT 3'
rat Krt5	5' AGG AGC AGG CAG TGG ATT C 3'
	5' GTT CAG GGG TGT GAG GAG ATT 3'
rat Krt14	5' TGG TTG GCA GTG AGA AAG TG 3'
	5' GGT CTT GAA GTA GGG GCT GTA G 3'
rat Krt10	5' GTA CGA GAA GCA CGG CAA CT 3'
	5' CAG GAC ATT GGC ATT GTC AG 3'
rat TERT	5' ATG TTC CTG TTC TGG CTA ATG 3'
	5' GCT TGC TCC ACA CAC TCT TAC 3'
rat CD34	5' GCT CTC TGC CTG ATG AGT CT 3'
	5' TGG TGT GGT CTT ATT GCT ATC T 3'
rat ITGA6	5' GAA GTT GGT GGA GAG ACT GA 3'
	5' CAA GGA GAA GAT GTG CTG AC 3'
rat Itgb1	5' ATG GGA AAC TTG GTG GTA TTG 3'
	5' ACA GGC TGG AAC TCT TCA GTG 3'
rat Sox9	5' CTT GGC TCC TTC AGA GTT AGT 3'
	5' AAT CCC CTC AAA ATG GTA AT 3'
rat Krt19	5' ATT CCA CAC CAG GCA TTG AC 3'
	5' TCC GTG ACC TCA GTC TTG TTT 3'

rat b-actin	5' CCT CTA TGC CAA CAC AGT 3'
	5' AGC CAC CAA TCC ACA CAG 3'

Supplementary table 2. siRNA seq

Target gene	SEQ
Human cdh1	GCCAGGAAATCACATCCTA
Rat cdh1	CAGCACGTATACAGCTCTCA
HUMAN FOXO1	CCGCGCAAGAGCAGCTCGT
RAT FOXO1	TCCAAACACCAGTCTAAATT
HUMAN KLF4	GACCGAGGAGGGCAACGAG
RAT KLF4	CACCGGGCCGGACACAGGA

Supplementary table 3. CHIP-SEQ primer seq

Target gene	Primer sequence
RAT Klf4	5' GGT CTT GGT GTG CTG GAT AA 3'<br>5' CTG AAA AAA ACA CGG CAT CTT 3'
RAT Krt5	5' AGG GAG TGT GCT GAA CTA AA 3'<br>5' GAG AAC AAT AGG GAC AAG AGT G 3'
RAT Krt14	5' GGC TCT GAG AAG TAC CTG ACT AT 3'<br>5' TAT TCA CCA TAC ACC TAC ACA CA 3'
RAT Krt15	5' GGG AGC AAG CAA AGG TTT AGT 3'

	5' GAT TGG GCT GAC AGG AGT CT 3'
RAT Krt19	5' GGG CAT AGG ATG TCT GTC T 3'<br><br>5' TGA TAC TCA CTA CCA GGA CAC T 3'
